# Do demographic and clinical features and comorbidities affect the risk of spread to an additional body site in functional motor disorders?

**DOI:** 10.1007/s00702-022-02537-x

**Published:** 2022-08-16

**Authors:** Tommaso Ercoli, Michele Tinazzi, Christian Geroin, Enrico Marcuzzo, Roberto Erro, Sofia Cuoco, Roberto Ceravolo, Sonia Mazzucchi, Andrea Pilotto, Alessandro Padovani, Luigi Michele Romito, Roberto Eleopra, Mario Zappia, Alessandra Nicoletti, Carlo Dallocchio, Carla Arbasino, Francesco Bono, Giorgio Spano, Benedetta Demartini, Orsola Gambini, Nicola Modugno, Enrica Olivola, Laura Bonanni, Alberto Albanese, Gina Ferrazzano, Alessandro Tessitore, Leonardo Lopiano, Giovanna Calandra-Buonaura, Martina Petracca, Francesca Morgante, Marcello Esposito, Antonio Pisani, Paolo Manganotti, Lucia Tesolin, Francesco Teatini, Fabrizio Stocchi, Giovanni Defazio

**Affiliations:** 1 Department of Medical Sciences and Public Health, University of Cagliari, Cagliari, Italy; 2 Neurology Unit, Movement Disorders Division, Department of Neurosciences, Biomedicine and Movement Sciences, University of Verona, P.le Scuro 10, 37134 Verona, Italy; 3 Center for Neurodegenerative Diseases (CEMAND), Department of Medicine, Surgery and Dentistry, Scuola Medica Salernitana, University of Salerno, Baronissi, SA Italy; 4 Center for NeuroDegenerative Diseases Parkinson and Movement Disorders, Department of Clinical and Experimental Medicine, University of Pisa, Pisa, Italy; 5 Neurology Unit, Department of Clinical and Experimental Sciences, University of Brescia, Brescia, Italy; 6 FERB Onlus, Ospedale S. Isidoro, Trescore Balneario, Bergamo Italy; 7 Parkinson and Movement Disorders Unit, Fondazione IRCCS Istituto Neurologico Carlo Besta, Milan, Italy; 8 Department G.F. Ingrassia, Section of Neurosciences, University of Catania, Catania, Italy; 9 Department of Medical Area, Neurology Unit, ASST Pavia, Pavia, Italy; 10 Botulinum Toxin Center, Neurology Unit A.O.U. Mater Domini, Catanzaro, Italy; 11 Aldo Ravelli Research Center for Neurotechnology and Experimental Brain Therapeutics, Department of Health Sciences, University of Milan, Milan, Italy; 12 IRCCS Neuromed, Pozzilli, Italy; 13 Department of Neuroscience, Imaging and Clinical Sciences, University G. d’Annunzio, Chieti-Pescara, Italy; 14 Department of Neurology, IRCCS Humanitas Research Hospital, Rozzano, Milan Italy; 15 Department of Human Neurosciences, Sapienza, University of Rome, Rome, Italy; 16 Department of Advanced Medical and Surgery Sciences, University of Campania-Luigi Vanvitelli, Naples, Italy; 17 Department of Neuroscience-Rita Levi Montalcini, University of Turin, Turin, Italy; 18 Department of Biomedical and Neuromotor Sciences, University of Bologna, Bologna, Italy; 19 IRCCS Istituto delle Scienze Neurologiche di Bologna, Bologna, Italy; 20 Movement Disorder Unit, Fondazione Policlinico Universitario A. Gemelli IRCCS, Rome, Italy; 21 Neurosciences Research Centre, Molecular and Clinical Sciences Neurosciences Research Centre, Molecular and Clinical Sciences Research Institute, St George’s University of London, London, UK; 22 Department of Experimental and Clinical Medicine, University of Messina, Messina, Italy; 23 Clinical Neurophysiology Unit, Cardarelli Hospital, Naples, Italy; 24 IRCCS Mondino Foundation, Pavia, Italy; 25 Department of Brain and Behavioral Sciences, University of Pavia, Pavia, Italy; 26 Clinical Neurology Unit, Department of Medical, Surgical and Health Services, University of Trieste, Trieste, Italy; 27 Functional Movement Disorders Outpatient Clinic, Clinical Neurology and Stroke Unit, Central Country Hospital, Bolzano, Italy; 28 University and Institute of Research and Medical Care San Raffaele Roma, Rome, Italy

**Keywords:** Functional neurological disorders, Functional motor disorders, Phenotypic change, Spread, Outcome

## Abstract

The aim of this study is to assess changes in the body distribution and the semeiology of functional motor disorder (FMD) in patients who reported only one or more than one body site affected at FMD onset. Data were obtained from the Italian Registry of Functional Motor Disorders, which included patients with a diagnosis of clinically definite FMDs. The relationship between FMD features and spread to other body sites was estimated by multivariate Cox regression analysis. We identified 201 (49%) patients who reported only one body site affected at FMD onset and 209 (51%) who reported multiple body sites affected at onset. FMD spread from the initial site to another site in 43/201 (21.4%) patients over 5.7 ± 7.1 years in those with only one site affected at FMD onset; FMD spread to an another body site in 29/209 (13.8%) over 5.5 ± 6.5 years. The spread of FMD was associated with non-motor functional symptoms and psychiatric comorbidities only in the patients with one body site affected at FMD onset. Our findings provide novel insight into the natural history of FMD. The number of body sites affected at onset does not seem to have a consistent influence on the risk of spread. Furthermore, our findings suggest that psychiatric comorbidities and non-motor functional symptoms may predict the spread of FMD symptoms, at least in patients with one body site affected at onset.

**Supplementary Information:**

The online version contains supplementary material available at 10.1007/s00702-022-02537-x.

## Introduction

Functional motor disorders (FMDs) manifest with involuntary movements, weakness or gait disorders, which are typically inconsistent and incongruent with recognized neurological diseases (Carson et al. 2012; Hallett et al. 2022; Lidstone et al. 2022; Stone et al. 2020). Such inconsistency implies that the clinical features of FMDs may change over time (Ercoli et al. 2021; Gupta and Lang 2009).

Phenotypic changes may be categorized by body distribution and semeiology of core functional motor symptoms. Theoretically, under the criterion of body distribution, the changes in a FMD pattern may be characterized by replacement of the affected body site or spread to additional body sites; under the criterion of semeiology of core functional motor symptoms, a FMD pattern may be classified as unchanged (the same symptom is present at subsequent follow-up) or a switch to or the addition of a phenomenologically different motor disorder.

Phenotypic changes may variably combine over time and contribute to an apparently unpredictable clinical heterogeneity that may render standardized disease pathways difficult to track (Espay et al. 2018). Focusing on just one or a few phenomenological aspects rather than on the overall clinical complexity of FMD would probably aid in differentiating the clinical heterogeneity within and among patients and make the natural history pathways of pathophysiological and/or prognostic relevance easier to identify.

In their recent study, Tomic and colleagues (2020) focused on semeiological changes over time alone (including additional FMDs and/or onset of non-motor functional symptoms) and reported that such changes were more likely to occur in patients who presented with FMD other than dystonia and a higher level of somatoform experience at baseline. They did not analyze changes in the spatial distribution of FMD, however.

With the present study, we wanted to determine whether the spatial distribution of FMD over time reflects its natural history. To do this, we studied the changes in body distribution and semeiology in patients who reported only one or more than one body site affected at FMD onset.

## Methods

Data were obtained from the Italian Registry of Functional Motor Disorders (IRFMD) (Tinazzi et al. 2020a) which includes patients with a diagnosis of clinically definite FMDs based on Gupta and Lang’s diagnostic criteria (Gupta and Lang 2009). The IRFMD is a multicenter initiative that includes 25 Italian centers coordinated by the Italian Academy for the Study of Parkinson’s Disease and Other Movement Disorders (Accademia LIMPE-DISMOV RADAC project) and Fondazione LIMPE. As for other Italian registries (Defazio et al. 2020a; Tinazzi et al. 2020b), patients’ information is recorded into a web-based encrypted anonymized system in the web site of the Accademia LIMPE-DISMOV (https://www.accademialimpedismov.it/radac). Approval was obtained from the Institutional Ethics Committee of the Coordinating Centre (University of Verona, Azienda Ospedaliera Universitaria Integrata Verona, Prog. 1757CESC) and confirmed by the Committees of each participating center. The main demographic and clinical features of the patients included in the registry have been described elsewhere (Tinazzi et al. 2020a). The IRFMD contains demographic/clinical information on age, sex, education, phenotype (tremor, weakness, dystonia, jerks, gait disorders), and affected body region (cranial, cervical, one or both upper limbs, one or both lower limbs, trunk/abdomen) on examination, and timing (year of onset) of FMD in each body site (Tinazzi et al. 2021a; b).

We stratified the study population according to the number of affected body sites at FMD onset: (i) patients with one body site affected at disease onset; (ii) patients with multiple body sites affected at disease onset. We did not take into consideration a history of FMD but not evident at the time of the present study because a diagnosis of FMD based on our own observation could not be established.

The IRFMD also provided information about potentially associated factors, e.g., additional non-motor functional symptoms (sensory symptoms, dissociative seizures, visual symptoms, cognitive disorders, fibromyalgia, irritable bowel syndrome), neurological comorbidities (migraine, parkinsonism, neuropathy, hyperkinetic movement disorders, seizures, multiple sclerosis, cerebrovascular diseases, lumbar back pain, carpal tunnel syndrome, spinal disc herniation), psychiatric comorbidities (anxiety, major depression, somatoform disorders), and disease modeling (family history of neurological diseases, friends with neurological diseases). Information on associated factors were supported by informed relatives and medical records.

Statistical analysis was performed with the Stata 11.0 package (StataCorp LP, College Station, TX, USA). Data are expressed as mean ± standard deviation (SD) unless otherwise indicated. Differences between groups were tested using the chi-square test, Fisher’s exact test or the Mann–Whitney *U* test, as appropriate. The relationship between FMD features and spread to other body sites was estimated by Cox regression analysis. Study time was defined as the time between FMD onset and spread to another body site; patients in whom spread never occurred were included in the survival functions for the duration of observation. Cox proportional hazard regression analysis was adjusted for age and years of schooling as continuous variables and for female sex as a categorical variable (1 if present, 0 if not). Hazard ratio (HR), two-sided 95% confidence interval, (95% CI), and *p* value were computed (Defazio et al. 2020b). Significance was set at 0.05 and adjusted by Bonferroni correction, as needed.

## Results

We identified 201 (49%) of the 410 patients in the IRFMD (as of August 2019) who reported only one body site affected at disease onset and 209 (51%) patients who reported more than one site affected at onset. The two groups were similar for age, sex, education, disease duration, frequency of dystonia, acute onset, other functional non-motor disorders, psychiatric and neurological comorbidities, disease modeling, and spread to another body site (Table 1). Differently, the patients with multiple body sites affected at onset reported a higher frequency of tremor, weakness, jerks, gait disorders, and a shorter time to spread of disease (Table 1).

**Table 1:** Demographic and clinical features of patients who experienced one or multiple body sites affected at disease onset

Variable	Patients with one body site affected at FMD onset (*n* = 201)	Patients with multiple body sites affected at FMD onset (*n* = 209)	*p* value
Women—no. (%)	144 (71.6)	147 (70.3)	0.82
Age—years, mean (SD)	45.7 ± 16.2	47.5 ± 15.3	0.11
Education—years, mean (SD)	11.7 ± 4	11.6 ± 3.9	0.79
FMD at onset—no. (%)			
Tremor	57 (28.4)	93 (44.5)	**0.001**
Weakness	61 (30.3)	103 (49.3)	** < 0.001**
Dystonia	40 (19.9)	59 (28.2)	0.05
Jerks	29 (14.4)	58 (27.8)	**0.001**
Gait disorders	26 (12.9)	77 (36.8)	** < 0.001**
Acute FMD onset—no. (%)	145 (72.1)	145 (69.4)	0.58
Non-motor functional symptoms—no. (%)	86 (42.8)	110 (52.6)	0.04
Psychiatric comorbidities—no. (%)	68 (33.8)	79 (37.8)	0.41
Neurological comorbidities—no. (%)	45 (22.4)	44 (21.1)	0.81
Disease modelling—no. (%)	41 (22.4)	64 (30.6)	0.02
Frequency of spread—no. (%)	43 (21.4)	29 (13.8)	0.05
Time to spread—years, mean (SD)	2.7 ± 2.2	2.1 ± 1.9	**0.0033**

Significance was set at 0.004 after Bonferroni correction

Values in bold indicate statistically significant results.

### *Patients with one body site affected at disease onset*

Among the 201 patients with only one body site affected at onset, FMD spread from the initial site to an additional site in 43 (21.4%) over 5.7 ± 7.1 years. In the 43 patients who experienced spread the time to spread was significantly shorter than the disease duration in the 158 patients who did not (2.7 ± 2.2 vs. 5.5 ± 7.5 years, *p* = 0.01). Univariate Cox analysis showed that sex, age, education, neurological comorbidities, disease modeling, and site or phenotype of FMD at onset were not significantly associated with spread (Table 2), whereas spread was closely associated with other non-motor functional symptoms and psychiatric comorbidities (Table 2). Multivariate Cox analysis adjusted for age, sex, and years of schooling confirmed the independent association between FMD and other non-motor functional symptoms (adjusted HR 2.01; 95% CI, 1.01–4.01; *p* = 0.04) and psychiatric comorbidities (adjusted HR 2.1; 95% CI, 1.07–4.1; *p* = 0.03). Only 27/201 (13.4%) patients reported changes in FMD semiology at subsequent follow-up. Most patients who reported changes in FMD semiology also experienced spread to an additional body site: changes in FMD semeiology were reported by 23/43 (53.4%) patients who also reported spread of FMD and by 4/158 (2.3%) who did not (*p* < 0.001).

**Table 2:** Demographic and clinical features of patients (*n* = 201) with only one body site affected at onset who reported spread to an additional body site and those who did not

Variable	Patients who reported spread (*n* = 43)	Patients who did not report spread (*n* = 158)	Hazard ratio	95% confidence interval	*p* value
Women—no. (%)	28 (65.1)	116 (73.4)	1.37	0.73–2.57	0.32
Age—years, mean (SD)	48.7 ± 14.3	44.9 ± 16.7	1.01	0.98–1.02	0.42
Education—years, mean (SD)	12 ± 3.1	11.7 ± 3.8	1.0	0.92–1.11	0.83
Site of onset—no. (%)					
Cranial region	8 (18.6)	16 (10.1)	1.71	0.79–3.71	0.17
Neck	3 (7)	11 (7)	0.99	0.31–3.23	1
Upper limb	15 (34.9)	61 (38.6)	0.88	0.47–1.64	0.69
Lower limb	17 (39.5)	66 (41.8)	0.94	0.51–1.74	0.86
Trunk/abdomen	0	4 (2.5)	–	–	1
FMD at onset—no. (%)					
Tremor	11 (25.6)	46 (29.1)	0.81	0.41–1.62	0.56
Weakness	12 (27.9)	46 (29.1)	1.07	0.55–2.08	0.84
Dystonia	6 (14)	28 (17.7)	0.72	0.32–1.63	0.44
Jerks	8 (18.6)	19 (12)	1.26	0.58–2.72	0.55
Gait disorders	6 (14)	19 (12)	1.11	0.46–2.64	0.81
Acute FMD onset—no. (%)	29 (67.4)	116 (73.4)	0.91	0.48–1.73	0.78
Non-motor functional symptoms—no. (%)	24 (55.8)	62 (39.2)	2.21	1.21–4.07	**0.01**
Psychiatric comorbidities—no. (%)	23 (53.5)	45 (28.5)	2.3	1.26–4.19	** < 0.01**
Neurological comorbidities—no. (%)	9 (20.9)	36 (22.8)	0.76	0.36–1.59	0.47
Disease modeling—no. (%)	13 (30.2)	28 (17.7)	1.9	0.98–3.65	0.06

Hazard ratio was estimated by univariate Cox analysis

Values in bold indicate statistically significant results.

### *Patients with multiple body sites affected at disease onset*

Among the 209 patients with multiple body sites affected at onset, FMD spread from the initial sites to an additional body site in 29 (13.8%) patients over 5.5 ± 6.5 years. In these 29 patients who experienced spread of disease, the time to spread was significantly shorter than the disease duration in the 180 patients who did not (2.1 ± 1.9 vs. 5.1 ± 6.4 years, *p* = 0.01). Cox regression analysis showed no significant differences in age, sex, years of schooling, phenotype of FMD at onset, acute FMD onset, non-motor functional symptoms, neurological and psychiatric comorbidities, and disease modeling between the 29 patients who reported spread and the 180 who did not (data not shown). Only 20/209 (9.6%) patients with multiple body sites affected at FMD onset reported changes in FMD at subsequent follow-up: such changes occurred in 20/29 (68.9%) who reported spread and 0/180 patients who did not (*p* < 0.0001).

## Discussion

These patients were stratified by the number of body sites affected at disease onset into two large groups that presented with FMD starting at one or more than one body site. The two groups were similar for several demographic and clinical features but differed for semeiology: tremor, weakness, jerks, and gait disorders were far more frequent in those with multiple body sites affected at FMD onset.

The spread of FMD was significantly associated with other non-motor functional symptoms and psychiatric comorbidities in the patients who reported it starting at one body site, whereas none of the variables was closely associated with the risk of spread in the patients who reported multiple body sites affected at FMD onset. In support of this view, previous studies suggested that psychiatric comorbidities and non-motor functional symptoms have prognostic relevance (Gelauff et al. 2014; Nicholson et al. 2020; Tomić et al. 2020). The varying association between other non-motor functional symptoms/psychiatric comorbidities and the risk of spread in the two groups of FMD patients might reflect pathophysiological differences, but a lack study power for the second group might also be responsible for the differential findings.

Our patients also experienced changes in FMD semeiology over time. This phenomenon was consistently more frequent among those who experienced spread to an additional body site, regardless of the number of body sites affected at onset. This would suggest a link between two potential markers of disease progression/prognosis. Since changes in FMD body distribution are less prone to recall bias and observer judgment than semeiological changes, the former may more reliably mark the course of FMD.

This study has several limitations. Although not a population-based study, a selection bias was unlikely because consecutive recruitment in multiple referral centers yielded a study cohort with clinical/demographic features similar to those observed in the general population of FMD patients. Bias due to the examiners being unblinded to the disease course was also unlikely, since clinical information was collected in a standardized fashion and the examiners were unaware of the study hypothesis. The comparable educational level of the patients who changed phenotype and that of the patients who did not rule out a recall bias. Owing to the retrospective assessment, prior possible FMDs that were however, not evident at study time were not considered by our analysis because we could not be confident on an FMD diagnosis that was not based on our own observation. Due to the lack of studies that systematically assess the frequency and the degree of overlap of various patterns of change in FMD over time, we were unable to know whether (1) the few patients who switched to a phenotypically different movement disorder and (2) the lack of patients reporting replacement of the initially affected body site with another site truly reflected a methodological bias inherent to the cross-sectional/retrospective design of the present study.

These limitations notwithstanding, our findings provide novel insight into the natural history of FMD. FMD may start in a single body site in about half of cases and then spread to other sites in 15–20% of cases. The number of body sites affected at onset does not seem to have a consistent influence on the risk of spread in frequency of and time to spread. Our findings suggest that psychiatric comorbidities and non-motor functional symptoms may predict the spread of FMD symptoms, at least in patients with one body site affected at onset. Further prospective long-term studies are needed to better answer the questions addressed in our study.

## Supplementary Information

Below is the link to the electronic supplementary material.

Supplementary file1 (DOCX 16 KB)

## Data Availability

Data are available upon reasonable request.
