# Supplementary material for: Do demographic and clinical features and comorbidities affect the risk of spread to an additional body site in functional motor disorders?
Source: J Neural Transm (Vienna). 2022 Aug 16;129(10):1271–6. doi: 10.1007/s00702-022-02537-x (PMC9468120; doi:10.1007/s00702-022-02537-x)
Supplement: Supplementary file 1 — Supplementary file1 (DOCX 16 KB) [file 702_2022_2537_MOESM1_ESM.docx]

**Supplementary Appendix. Co-investigators, Italian Registry of Functional Motor Disorders (IRFMDs) Study Group**

| **Name** | **Location** | **Role** | **Contribution** |
| --- | --- | --- | --- |
| Paolo Barone, MD, PhD | Center for Neurodegenerative Diseases (CEMAND) Department of Medicine, Surgery and Dentistry - Scuola Medica Salernitana, University of Salerno, Baronissi (Sa), Italy. | Site Investigator | Site coordinator of data acquisition |
| Sara Scannapieco, MD | Center for Neurodegenerative Diseases (CEMAND) Department of Medicine, Surgery and Dentistry - Scuola Medica Salernitana, University of Salerno, Baronissi (Sa), Italy. | Site Investigator | Site coordinator of data acquisition |
| Daniela Frosini, MD | Neurology Unit, Department of Clinical and Experimental Medicine, University of Pisa, Pisa, Italy. | Site Investigator | Site coordinator of data acquisition |
| Eleonora Del Prete, MD | Neurology Unit, Department of Clinical and Experimental Medicine, University of Pisa, Pisa, Italy. | Site Investigator | Site coordinator of data acquisition |
| Andrea Scalvini, MD | Department of Clinical and Experimental Sciences, University of Brescia, Brescia, Italy. | Site Investigator | Site coordinator of data acquisition |
| Alberto Imariso, MD | Department of Clinical and Experimental Sciences, University of Brescia, Brescia, Italy. | Site Investigator | Site coordinator of data acquisition |
| Antonio Emanuele Elia, MD | Parkinson and Movement Disorders Unit, Fondazione IRCCS Istituto Neurologico Carlo Besta, Milan, Italy. | Site Investigator | Site coordinator of data acquisition |
| Nico Golfrè Andreasi, MD | Parkinson and Movement Disorders Unit, Fondazione IRCCS Istituto Neurologico Carlo Besta, Milan, Italy. | Site Investigator | Site coordinator of data acquisition |
| Giovanni Mostile, MD, PhD | Department G.F. Ingrassia, Section of Neurosciences, University of Catania, Catania, Italy. | Site Investigator | Site coordinator of data acquisition |
| Antonina Luca, MD, PhD | Department G.F. Ingrassia, Section of Neurosciences, University of Catania, Catania, Italy. | Site Investigator | Site coordinator of data acquisition |
| Sara Mazza, MD | Department of Medical Area, Neurology Unit, ASST Pavia, Pavia, Italy. | Site Investigator | Site coordinator of data acquisition |
| Gabriele Bellavia, MD | Department Of Medical Area, Neurology Unit, ASST Pavia, Pavia, Italy. | Site Investigator | Site coordinator of data acquisition |
| Marilisa Pasquale | Botulinum Toxin Center, Neurology Unit A.O.U. Mater Domini, Catanzaro, Italy. | Site Investigator | Site coordinator of data acquisition |
| Pietro Antonio Bruno | Botulinum Toxin Center, Neurology Unit A.O.U. Mater Domini, Catanzaro, Italy. | Site Investigator | Site coordinator of data acquisition |
| Alberto Priori MD, PhD | Aldo Ravelli Research Center For Neurotechnology and Experimental Brain Therapeutics, Department of Health Sciences, University of Milan, Milan Italy. | Site Investigator | Site coordinator of data acquisition |
| Cinzia Femiano, MD | IRCCS Neuromed, Pozzilli, Italy. | Site Investigator | Site coordinator of data acquisition |
| Giada Ricciardo Rizzo, MD PhD | IRCCS Neuromed, Pozzilli, Italy. | Site Investigator | Coordinated acquisition of data for site |
| Marco Onofrj, MD | Department of Neuroscience, Imaging And Clinical Sciences -University G. D'annunzio, Chieti-Pescara, Italy. | Site Investigator | Site coordinator of data acquisition |
| Stefania Lalli, MD, PhD | Department of Neurology, IRCCS Humanitas Research Hospital, Rozzano, Milan, Italy. | Site Investigator | Site coordinator of data acquisition |
| Giovanni Fabbrini, MD | Department of Human Neurosciences, Sapienza, University of Rome, Rome, Italy.  IRCCS Neuromed, Pozzilli, Italy. | Site Investigator | Site coordinator of data acquisition |
| Rosa De Micco PhD | Department of Advanced Medical and Surgery Sciences, University of Campania - Luigi Vanvitelli, Naples, Italy. | Site Investigator | Site coordinator of data acquisition |
| Maurizio Zibetti, MD, PhD | Department of Neuroscience - Rita Levi Montalcini, University of Turin, Turin, Italy. | Site Investigator | Site coordinator of data acquisition |
| Luisa Sambati MD, PhD | Department of Biomedical and Neuromotor Sciences, University of Bologna, Bologna, Italy  IRCCS Istituto delle Scienze Neurologiche di Bologna, Bologna, Italia. | Site Investigator | Site coordinator of data acquisition |
| Anna Rita Bentivoglio, MD, PhD | Institute of Neurology, Movement Disorder Research Center, Università Cattolica del Sacro Cuore; Movement Disorder Unit, Fondazione Policlinico Universitario A. Gemelli IRCCS, Rome, Italy. | Site Investigator | Site coordinator of data acquisition |
| Giulia Di Lazzaro, MD | Department Systems Medicine, University of Rome Tor Vergata, Rome, Italy. | Site Investigator | Site coordinator of data acquisition |
| Giulia Bellavita, MD | Clinical Neurology Unit, Department of Medical, Surgical and Health Services, University of Trieste, Italy. | Site Investigator | Site coordinator of data acquisition |
